# Vitamin D and Cardiovascular Diseases: An Update

**DOI:** 10.7759/cureus.49734

**Published:** 2023-11-30

**Authors:** Farrookh Haider, Hashsaam Ghafoor, Omar F Hassan, Khalid Farooqui, Ali O. Mohamed Bel Khair, Faryal Shoaib

**Affiliations:** 1 Department of Internal Medicine, Section of Cardiology Al Khor Hospital, Hamad Medical Corporation, Al Khor, QAT; 2 Department of Internal Medicine, College of Medicine/Qatar University, Doha, QAT; 3 Department of Anesthesia, Al Khor Hospital, Hamad Medical Corporation, Al Khor, QAT; 4 Department of Anesthesiology, Qatar University, Doha, QAT; 5 Department of Internal Medicine, Al Khor Hospital, Hamad Medical Corporation, Al Khor, QAT; 6 Department of Internal Medicine, Shifa International Hospitals, Islamabad, PAK

**Keywords:** anesthesia considerations, supplements, cardiovascular diseases, deficiency, vitamin d

## Abstract

Vitamin D is a vital nutrient that plays a significant part in several physiological processes within the human body, including calcium metabolism, bone health, immune function, and cell growth and differentiation. It is obtained mainly through exposure to sunlight but can be acquired from certain foods and supplements as well. Vitamin D deficiency (VDD) could be the risk factor for cardiovascular diseases (CVDs), such as heart disease and stroke. In blood vitamin D low levels have been linked with an enhanced risk of developing CVDs. However, it is unclear whether vitamin D levels are the leading cause or consequence of these conditions. While some studies highlight that taking vitamin D supplements could decrease the risk of CVD; however, more research is required to better understand the association between vitamin D and cardiovascular health. In this review, we aimed to summarize the currently available evidence supporting the association between vitamin D and CVDs and anesthesia considerations.

## Introduction and background

Vitamin D (VD) is a fat-soluble vitamin that plays a significant role in several physiological processes within the human body. It is primarily known for its role in maintaining bone health by promoting the absorption of phosphorus and calcium from the diet, which are essential for bone growth and development [1-3]. VD is also involved in several other functions, including immune system regulation, cell growth and differentiation, muscle function, and cardiovascular health. VD is produced in the skin from exposure to sunlight and can be acquired from certain foods and supplements [4,5]. There are two major types of VD: D2 (ergocalciferol) and D3 (cholecalciferol). Vitamin D2 is mostly found in plant-based sources such as mushrooms, whereas vitamin D3 is produced in the skin in response to sunlight and found in animal-based sources such as egg yolk and fatty fish [6,7].

Although VD deficiency (VDD) is rare in healthy individuals with enough sun exposure and a balanced diet, it can occur in individuals with limited exposure to sunlight, darker skin color, or certain medical disorders affecting VD metabolism. VDD is linked to an enhanced risk of several health conditions including bone disorders, autoimmune diseases, and cardiovascular disease (CVD) [8-10]. As such, it is important for individuals to maintain sufficient levels of VD through the intake of a balanced and varied diet, moderate exposure to sunlight, and, in some cases, VD supplementation. However, before starting any supplement, excessive VD intake can have harmful effects on health. A growing interest has recently been observed in the possible role of VD in the avoidance of CVD [11].

## Review

VD physiology and metabolism

Once VD is consumed, it is assimilated into chylomicrons that are then absorbed by the lymphatic system and enter into circulation [12-15]. During digestion, vitamin D2 is bound to a VD-binding protein (VDBP) and albumin [16,17]. VD is transferred to the liver, where it is transformed into 25(OH)D via vitamin-D-25-hydroxylase [15]. Within the kidney, it is transformed into 1,25(OH)2D via 25-hydroxyvitamin D-1α [15,18]. 1,25(OH)2D binds to VDBP in the blood, and then upon disassociation, it binds to the intracellular nuclear VD receptor (VDR) to wield several physiologic functions while maintaining its own levels [14,15]. Although 1,25-hydroxycholecalciferol and 25-hydroxycholecalciferol are mainly produced in the kidneys and liver, respectively, in the epidermis, the keratinocytes express 25-hydroxylase(CYP27A1) [19], 1,25-hydroxycholecalciferol, and 1α-hydroxylase(CYP27B1) [20]. However, the skin’s ability to synthesize 1,25(OH)2 D3 is rare [21] because the skin itself is the target tissue [22]. As such, VD and its complex interaction with calcium encourage keratinocyte differentiation [23].

Although the highest concentrations of the VDR exist within cells engaged in calcium homeostasis [14,24], VDR is found in all cells of the human body [13,14,24,25]. Several cells also perform 25(OH)D1 alpha-hydroxylase action (CYP27B1) that enables 1,25(OH)2D elimination through the kidneys. The enzyme in these tissues is controlled by certain factors-for example, stage of cell development and provocative signaling molecules. In addition, the extra-renal tissues can degrade 1,25(OH)2D [14]. The activation of VDR by 1,25(OH)2D causes massive biological activations within these tissues through both genomic and nongenomic pathways [13,14].It is known VD boosts the immune system and possesses sturdy immunomodulatory capability. Numerous components associated with VD are found in the cardiovascular system, signifying it plays a significant role in cardiovascular health. The extraskeletal functions of VD are currently being measured as targets to decrease the risk of carcinoma, neurocognitive dysfunction, autoimmune and other diseases, and death [14]. However, additional research is required [26].

VD testing

The plasma/serum level of 25(OH)D is a normally accepted biological marker of VD status. Regarding several other metabolites, 25(OH)D reflects appropriate dietary VD status, having the longest half-life of between two and three weeks. Vitamin D3 (cholecalciferol) has a half-life of 24 hours, and 1,25(OH)2D has a half-life of only four hours [27].

In circulation, the elevated percentage of 1,25(OH)2D and 25(OH)D bound to VDBP is 80% to 90%, whereas, for albumin, it is 10% to 20%, although an insignificant amount is not bound (0.2% to 0.6% of 1,25(OH)2D and 0.02% to 0.05% of 25(OH)D) [27]. No agreement exists on the ideal levels of 25(OH)D, according to two official reports issued by the Institute of Medicine [28] and the US Endocrine Society [29]. A concentration of 25(OH)D above 30 ng/mL is appropriate, as per the US Endocrine Society guidelines, whereas the cut-off value provided by the Institute of Medicine is 20 ng/mL [30].

Several techniques are used to test serum 25(OH)D. Liquid chromatography-tandem mass spectrometry is the gold standard because it can distinguish 25(OH)D2 and 25(OH)D3. Two techniques, radioimmunoassay and chemiluminescence, are extensively used along with the enzyme-linked immunosorbent assay [27]. The recommended dietary intake daily is between 400 and 1,000 IU [29]. Vitamin D2 (ergocalciferol) is the presumed measure in the diet, different from vitamin D3 (cholecalciferol) because of its insignificant affinity for VDBP. This is why supplementations do not lead to elevated VD serum concentrations within the blood [30].

VD and the cardiovascular system

**Figure 1:**
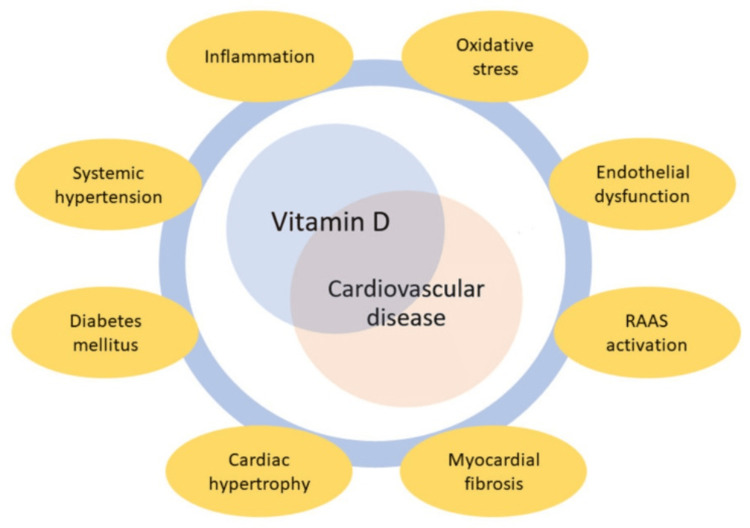
Major hypothetical mechanisms underlying the association between vitamin D and cardiovascular disease RAAS = renin-angiotensin-aldosterone system. With permission from [32].

The effects of VD on cardiovascular health can be measured by its emerging and classic cardiovascular risk determinants or its direct effects on the cardiovascular system [31]. An increasing volume of evidence has emphasized the association between VD and the cardiovascular system (Figure 1).

Fibroblasts, vascular endothelial and smooth muscle cells, and cardiomyocytes express the VDR and 1α-hydroxylase which is present in these cells , facilitating the production of active form of VD [33]. It has been demonstrated by experimental models that VD has numerous cardiovascular effects, such as antihypertrophic properties, cardiomyocyte proliferation inhibition, stimulation of proliferation of smooth muscle cells, endothelial growth factor expression, and inhibition of natriuretic peptide emission and the renin-angiotensin-aldosterone system [34]. The activation of VDR through calcitriol or its analogs can directly hinder the expression of angiotensin I and the local production of angiotensin II in the myocardium, kidney tissue, and renal arteries [35]. Furthermore, VD is found to stimulate the expression of angiotensin-converting enzyme-2, which is the enzyme that breaks down angiotensin II into angiotensin 1-7. It further counteracts the excessive level of angiotensin II and, therefore, fosters the antihypertensive, antifibrotic, and anti-inflammatory functions of angiotensin 1-7 [36]. Finally, the immune cells requiring VDR can have a direct effect on miR-106b-5p emissions. This could enhance renin production by acting upon juxtaglomerular cells, hence implying that inflammation is a major factor regarding renin-driven hypertension [37].

In addition, VD controls the expression of specific metalloproteinases and metalloproteinase inhibitors that encourage the development of heart failure [38]. Despite the direct impact, VD could have an indirect impact on the cardiovascular system via its influence on the cardiovascular risk determinants. The deficiency of VD is related to the development of hypertension [31,39], Type 2 Diabetes Mellitus, and dyslipidemia [31,40]. Finally, there is growing evidence highlighting the anti-inflammatory role it plays in hindering the activation of tumor necrosis factor-alpha and nuclear factor kappa B, as well as by promoting interleukin-10, which are all recognized as significant contributors to CVD [41]. Overall, these observations significantly support VD involvement in the progression as well as development of CVD and its possible effect on short- and long-term cardiovascular consequences [32].

Atherosclerosis

Atherosclerosis occurs due to an interplay between hereditary and environmental factors. The most common determinants that encourage the development of atherosclerosis are elevated low-density lipoproteins (LDLs) in the bloodstream, as well as hypertension [42]. Currently, various findings demonstrate an association between VD and atherosclerosis plaque development, probably mediated through immune response modulation [43]. These interactions are elucidated through the expression of VDR, CYP27B1, and CYP27A1 hydroxylases in immune cells. VD activation is linked to the latter, which plays an important role as a paracrine/autocrine modulator for atherosclerotic plaque pathobiology [44]. Active VD affects immune cells such as macrophages and dendritic cells. VD specifically induces the transition of monocytes into macrophages, resulting in cellular obligation toward the M1 phenotype. The M1 phenotype is accountable for generating immunosuppressant cytokines, such as prostaglandin E2, and for suppressing the expression of Toll-like receptors (TLR) such as TLR2, TLR4, and TLR9. As a result of the downregulation of class II main histocompatibility complex antigens at the cell surface, there is a reduction in pro-inflammatory cytokines synthesis [45].

VD has a regulatory effect on adaptive immune cells, specifically on B lymphocytes, inhibiting their propagation, their development into plasma cells, and immunoglobulin production [46]. Regarding T lymphocytes, VD represses the pro-inflammatory responses, dependent upon Th1 and Th17, while promoting the immunomodulatory activities of Th2, Treg, and Tr1 [47]. In addition, a study revealed the interaction between VD and VDR signaling can decrease scavenger receptor expression on macrophage surfaces in individuals with diabetes. This mechanism can prevent the accumulation of LDL cholesterol in foam cells, ultimately mitigating the risk of vascular atherosclerosis [48]. VDR activation also triggers an anti-atherosclerotic mechanism by inhibiting the expression of nuclear factor kappa-light-chain-enhancer of the activated B cells (NF-κB) gene. This results in the downregulation of pro-inflammatory as well as prothrombogenic cytokines, such as interleukin-6, and the upregulation of interleukin-10 and thrombomodulin. By inhibiting foam cell formation, this modulation of endothelial function suppresses vascular calcifications and thwarts the formation of atherosclerotic plaques [49]. Another study, conducted on pigs, indicated how sufficient intracellular concentrations of VD can downregulate NF-κB expression by preventing kariopherin-A4 from stimulating NF-κB expression [50]. ­­The major characteristics of these paracrine/autocrine pathways depend upon their independence from systemic levels of PTH, calcium, and 1,25-dihydroxylcalciferol. Hence, the lack of dependence on classical regulatory mechanisms may account for the inconclusive findings from clinical trials, assessing the cardiovascular effects of VD supplementation. Taken together, these factors justify the observed association between VDR expression and the burden of atherosclerotic plaques in vivo. Among transgenic rats overexpressing 24-hydroxylase, which deactivates 1,25-OH VD, marked atherosclerotic lesions were identified [51]. Furthermore, assessments have been carried out on human atherosclerotic plaques acquired from patients experiencing endarterectomy from carotid stenosis. These experimental annotations showed intraplaque VDR levels are associated with M1 phenotype macrophage expression. Differently from plasma VD levels, low levels of VDR within the downstream sections of carotid plaques envisage the risk of major adverse cardiovascular events (MACE) [52]. The participation of VD in the regulation of visceral, as well as ectopic fat deposition, may also contribute to cardiometabolic dysfunction. The outcomes of some clinical studies suggest concurrent supplementation of VD and calcium could reduce fat deposition, as well as the risk of cardiovascular and metabolic anomalies [53].

Stroke

Malnutrition is commonly observed among poststroke patients admitted to rehabilitation units, and this can have an impact on their nutritional indices and vitamin levels. As a result, strokes are considered the primary cause of population disability. VDD appears to have implications that go beyond the risk and severity of stroke, as it also affects the process of poststroke recovery [27].

Poole et al. reported a decreased level of 25(OH)D (below 20 ng/mL) among 77% of 44 patients investigated for 30 days after their first stroke. They postulated that VDD was possibly present before stroke, given that the biological half-life of 25(OH)D is almost three weeks and a severe decrease in 25(OH)D seemed unfeasible for the time between 25(OH)D sampling and the stroke event [54]. Liu et al. asserted a novel strategy to prevent stroke is the administration of VD. A VD supplement is aimed at conserving a 25(OH)D serum level of more than 30 ng/mL among patients following stroke insult and among people at an elevated risk of stroke. This supplement has been proposed to be a protective and promising treatment [55].

Pilz et al. highlighted the potential pathways linking VDD with enhanced risk of stroke. Arterial hypertension is a major risk factor regarding stroke and is related to low concentrations of vitamins; the mechanism appears to be associated with the renin-angiotensin-aldosterone system; the prevention of renoprotective, anti-inflammatory, and hyperparathyroidism effects; and vasculoprotective properties [56]. Furthermore, VDD is related to an enhanced risk for T2DM. Atherosclerosis, secondary hyperparathyroidism, inflammation, and prothrombotic states are among the other risk determinants for ischemic cerebrovascular incidents [57].

Muscogiuri et al. investigated the role of VDD in stroke, myocardial infarction, and atherosclerosis, describing the mechanism, along with a comprehensive description of research regarding other animal models [58]. A large-scale study conducted in Copenhagen and a meta-analysis by Brøndum et al. assessed a total of 10,170 respondents. During the 21-year follow-up period, 1,256 respondents developed ischemic stroke. When comparing the participants with acute VDD (10 ng/mL) with respondents to those with optimal VD levels (30.0 ng/mL), the multivariate-adjusted hazard ratio of ischemic stroke was 1.36 (1.09-1.70). The concentrations of VD were not correlated with hemorrhagic stroke risk. The results of this study were verified by a meta-analysis in which the lowest versus highest quartile of 25(OH)D was compared; the multivariate-adjusted odds ratio for ischemic stroke was 1.54 (1.43-1.65), along with a matching hazard ratio of 1.46 (1.35-1.58) in prospective studies [59].

A meta-analysis and systematic review of 191 studies were carried out by Zhou et al. to investigate the potential relationship of VD with stroke risk. The authors concluded low VD levels are related to an enhanced risk of ischemia, but not stroke or hemorrhage [60]. One study included in this meta-analysis demonstrated a correlation between VDD and stroke risk, regardless of race [61].

Ludwigshafen’s Risk and Cardiovascular Health study examined >3,000 patients for eight years, reporting that low concentrations of 25(OH)D and 1,25(OH)2D were prognostic of lethal stroke [62]. Another study on vitamins as stroke forecasters among aging people demonstrated that low intake of VD and a low concentration of 1,25(OH)2D were significant risks for stroke [63]. Several observational studies have suggested VD may protect people from stroke, but another study carried out among 25,871 participants reported that vitamin D3 (200 IU daily) supplements do not decrease cardiovascular incidents, including stroke [64].

Diabetes

Type-1 diabetes mellitus (T1DM) is caused by autoimmune obliteration of the pancreatic beta cells, causing complete insulin production deficiency [8]. Regarding type-2 diabetes mellitus development, the main mechanisms concerned are the dysfunction of beta cells, systemic inflammation, and peripheral insulin resistance. Based on the evidence, VDD is related to all these functions [65].

VD may exert effects on beta cell activity by directly associating with VDR receptors and through enzyme 1α-hydroxylase local expression. In addition, VD may potentially boost insulin sensitivity by stimulating VDR expression within peripheral tissues, as well as by triggering proliferator-activated receptor-gamma receptors in peroxisomes, which play a considerable part in controlling fatty acid metabolism in adipose tissue and skeletal muscle. In contrast, VD may contribute via indirect pathways to insulin emission and sensitivity by regulating the concentration of calcium and altering peripheral tissue and beta cell membranes [65].

Several studies have demonstrated that the prevalence of T1DM is found to be elevated among those countries within higher latitudes, and the disease is mostly identified during winter [66]. Many studies have linked VDD among pregnant women with the prevalence of T1DM among children after delivery [67]. Other research has assessed VD supplementation’s protective role during early infancy against the development of T1DM, indicating an insignificant incidence of diabetes among children given vitamin supplementation [68].

Regarding type-2 diabetes mellitus and insulin resistance, the outcomes have been contradictory. Several studies have linked low levels of 25-hydroxyvitamin D with insulin resistance and pancreatic beta cell dysfunction among Western populations [69]. However, a study carried out in 1807 healthy people among the Korean population by Ock et al. highlighted that there is an inverse relationship between insulin resistance and VD [70]. Analyzing the association between diabetes mellitus, coronary artery disease (CAD), and VDD, Nardin et al. assessed 1,859 patients experiencing elective angiography for CAD evaluation and determined that diabetes is an independent prognosticator of VDD; however, diabetic patients with VDD demonstrated enhanced CAD prevalence, as well as severity [71]. Schafer et al. followed more than 5,000 elderly women for 8.6 ± 4.4 years to explore a probable correlation between the concentration of VD and the frequency of type-2 diabetes mellitus and found no correlation between concentrations of VD and frequency of T2DM [72].

Hypertension

The inequity between vasodilatation and vasoconstriction caused by several genetic and epigenetic factors (VDD) leads to vasoconstriction causing significant hypertension (HTN) [73]. Among VDD patients who have hypertension, Chen et al. reported an important clinical reduction in blood pressure (BP) caused by VD’s antihypertensive effect [74]. Likewise, considerably low levels of VD are related to nondipper hypertension when compared with dipper hypertension [75]. Hypertensive individuals with a nocturnal decrease of 10% in mean daytime systolic and diastolic BP are categorized as nondippers and from 10 to 20% as dippers. In addition, an impaired renin-angiotensin system is an important risk determinant for hypertension, whereas low concentrations of VD are correlated with an impaired renin-angiotensin system (RAS). Negative regulation of RAS with VD supplementation signifies VD’s advantageous role in hypertension treatment [76]. Decreased vessel complication is caused by aortic stiffness, whereas atherosclerosis is related to hypertension. The relationship between low circulation VD and aortic firmness is independent of classical risk determinants and provocative indicators among prediabetic subjects, suggesting VDD is an important risk factor for hypertension and CVD [77]. However, Kang et al. highlighted that the relationship between the levels of VD and several health indicators, namely BP, glycemic index, lipid profiles, wall thickness of the intima media of the carotid artery, and brachial ankle pulse wave velocity, is influenced by gender, whereas VD serum level may not be a leading risk factor associated with arterial stiffness and subclinical atherosclerosis [78].

VD controls endothelial and vascular smooth muscle cell proliferation [79], and the VDR is found within these cells [80]. An important role is played by endothelial dysfunction in vascular diseases such as hypertension. VDD affecting the endothelial cells could precipitate HTN [81]. Damage to acetylcholine-induced aortic relaxation, enhanced sensitivity to angiotensin II hypertensive effects, and enhanced expression of the angiotensin II infusion-induced hypertrophy-sensitive myocardial genes within endothelial-specific VDR knockout mice, when compared to the control mice, recommend the endothelial VDR as a possible role in endothelial cell function and BP control. VDR agonists also play a therapeutic role in the management of endothelial cell dysfunction-associated CVD [82].

Coronary artery disease

Coronary artery disease incidence has been confirmed to relate to VDD; however, the pathophysiological systems of such a relationship have not yet been recognized [8]. The main indication for a possible relationship is the existence of VDR in the myocardium, vascular endothelial cells, and fibroblasts as well as a demonstration through epidemiological studies that the prevalence of both VDD and CAD are enhanced by activating RAAS and by increasing anti-inflammatory and decreasing proinflammatory mediators [83].

VDD occurs most commonly among acute myocardial infarction (AMI) cases, whereas preliminary studies highlight a probable relationship between VD and AMI diagnosis both in the short and long run [83]. Furthermore, VDD appears to increase due to repeated unfavorable cardiac events caused by its relationship with several affected blood vessels, cardiac remodeling, and AMI problems [84]. A study was carried out among 18,225 male patients, who were followed for 10 years. The results showed a correlation between low levels of VD and enhanced risk of AMI, even after adjusting for other risk factors [85]. In addition, some prospective studies found an elevated incidence of VDD among patients with AMI admitted to the hospital. Another study carried out among 239 patients with acute coronary syndrome (ACS) demonstrated that 96% of individuals were admitted to the hospital with low levels of VD [86].

Several studies demonstrated a possible independent relationship between acute VDD and intrahospital death among ACS patients. Correia et al. conducted a study examining 206 patients with ACS. They observed that patients who had low levels (<10 ng/mL) of VD had a 24% incidence of intrahospital cardiovascular death, which was considerably more than that seen among the remaining proportion of patients (4.9%) [87].

Regardless of these annotations, no recent conclusive results are available supporting the advantage of VD supplementation as an important strategy regarding cardiovascular safeguards in CAD [12,88]. On the one hand, very limited and contradictory data are available for VD supplementation during primary prevention [89,90]. On the other hand, the possible advantages of VD administration during early-phase AMI have not yet been explored. Clinical studies specifically focusing on the advantage of VD supplementation among AMI patients with regard to long-term outcomes are deficient, and only some studies are continuing to investigate its effect on surrogate primary outcomes-for example, inflammation and left ventricular (LV) remodeling [32].

Acute myocardial infarction

VDD appears to increase due to repeated unfavorable cardiac events because it is related to postinfarction difficulties and cardiac makeover among AMI patients. Numerous systems could potentially underlie the link between the risk of AMI and VD . VD reduces plasma levels of renin, which in turn results in a decrease in renin-angiotensin-aldosterone system inhibitors [83].

In 1978, a preliminary report from Denmark explored VD levels among 75 patients who had stable angina (53 AMI patients and 409 controls). The results of the study showed VD levels were considerably lower among participants with AMI or angina than among the controls [91]. Another case-control survey carried out in 1990 demonstrated lower levels of VD among patients than controls. However, this difference was considerably more evident between winter and spring. Relative risk of AMI is reduced because rising VD quartiles demonstrate an inverse relationship between AMI risk and VD levels [92]. In addition, these statistics have been validated among more recent groups of people. A study was carried out in Framingham in which 1,739 participants were examined, revealing the incidence of significant cardiovascular events was 80% and 50% more than those with VDD and insufficiency, respectively [93]. Particularly, patients without a history of CAD and VD levels below 10 ng/mL underwent a hazard quotient of 1.8 for the development of primary cardiovascular incidents during a follow-up period of five years when compared to participants who had VD levels above 15 ng/mL. A study carried out among male patients reported that low levels of VD were correlated with an elevated risk of AMI, despite controlling for several other cardiovascular risk determinants, and at 10-year follow-up, respondents with normal concentrations (above 30 ng/mL) of VD had almost half the risk of AMI [85]. These results were verified by a large-scale study that compared the lowermost to uppermost baseline categories of circulating VD concentration, where the adjusted pooled relative risk for total cardiovascular incidents was 1.52 [94]. Hence, there is increasing evidence that VDD represents an innovative risk determinant regarding AMI [83].

In line with these epidemiological statistics, prospective studies have shown an elevated incidence of VDD among patients with AMI admitted to the hospital. A study carried out among 239 patients with ACS indicated 96% of them showed VD levels below 30 ng/mL on arrival to a health facility [86]. By this, Ng et al. reported 74% of patients with AMI demonstrated low levels of VD, and, among these patients, 36% had acute VDD [95]. Correia et al. highlighted the mean serum level of VD (18.5 ng/mL) among 206 patients with AMI (7% patients with STEMI) and found an acute deficiency among 10% of patients analyzed [87]. In addition, the same results were reported in two studies conducted by De Metrio et al. and Aleksova et al., who both asserted that, among AMI patients, the incidence of hypovitaminosis D was 89% and 68%, respectively [84,96].

Not only do low levels of VD seem to be a common independent risk factor regarding AMI, but they are also linked with poorer outcomes. Correia et al. offered initial evidence signifying a probable independent relationship between VDD and in-hospital death among patients with ACS. The patients with VD concentrations <10 ng/mL had a 24% cardiovascular death rate after hospital admission, which was more than the remaining proportion of patients (4.9%, with a relative risk of 4.3) [87]. Khalili et al., studying 139 patients with STEMI, also found a likely relationship between in-hospital elevated death and hypovitaminosis D [97]. However, the results of the study did not demonstrate a significant difference in the in-hospital death rate between patients with normal and low levels of VD [98]. More convincing data have been presented on clinical long-term implications regarding low concentrations of VD in AMI. However, a study performed among 1,259 ACS patients by Ng et al. reported that VD’s lowest quartile (below 7.3 ng/mL) was related to persistent major unfavorable cardiovascular events. Especially, the relationship mainly regarded readmission to the hospital for successive ACS or severe decompensated heart failure [95]. Consistent with these results, VD’s lowest quartile was an important prognosticator of one-year death [84]. VDD was yet again a marginal independent prognosticator for in-hospital death, possibly caused by the comparatively low in-hospital death rate of the study populace, and this was related to the elevated risk of various in-hospital unfavorable cardiac events. VD’s lowest quartile was related to an elevated prevalence of bleeding requiring blood transfusion, although similar baseline values of hemoglobin were reported. This is an important issue in the AMI setting, as effective antithrombotic treatment is the backbone of therapy, and bleeding and blood transfusions play a harmful role in outcomes. A relationship was also observed between the lowermost VD quartile and incidence of severe respiratory insufficiency [84].

**Figure 2:**
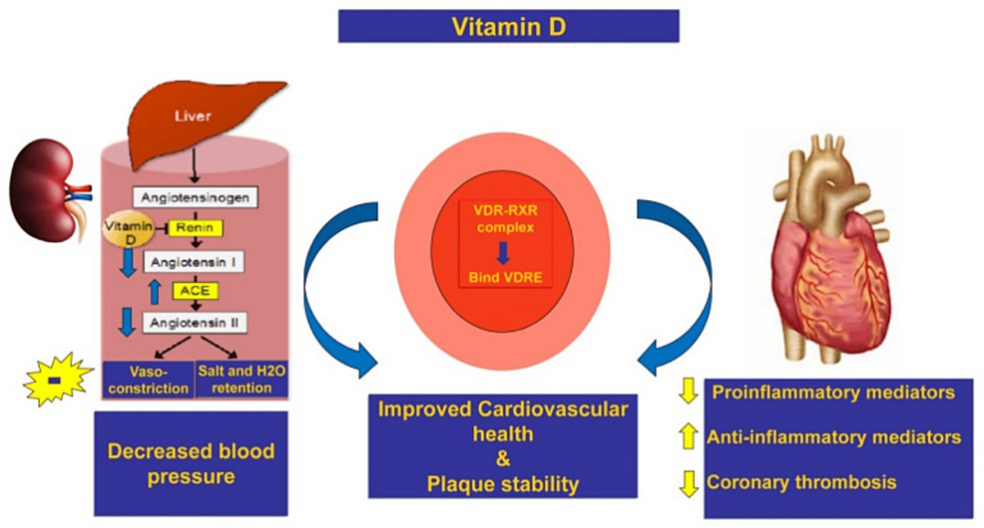
Role of vitamin D in cardiovascular protection when used with standard therapy for ACS patients through inhibition of inflammatory state, increased ACE as well as its antithrombotic and cardioprotective effects ACE-angiotensin- converting enzyme; ACS-acute coronary syndrome; VDR-vitamin D receptor; RXR-retinoid X receptor; VDRE-vitamin D response element. Arrows pointing up and down denote an increase and a decline, respectively; (-) indicates inhibition. With permission from [83].

The causal association between VD level and outcomes during AMI is yet to be explained. Among >3,000 patients experiencing coronary angiography, an important relationship between hypovitaminosis D and decreased LV function has been reported [99]. VDD has been reported to be related to mortality caused by sudden cardiac death and heart failure [100]. The myocardium, fibroblasts, and the vascular endothelium provide a platform for activation of VD via a complex interplay of VDR and 1-hydroxylase to the active form of VD. This in turn decreases angiotensin I levels, and subsequently, angiotensin II levels are decreased in myocardial and renal tissue. VD increases the levels of ACE2, which is responsible for converting excess angiotensin II into angiotensin I-7 and increased levels of angiotensin 1-7 result in potentiating the beneficial effects on fibrosis and inflammation leading to a decrease in BP. VDR acts as a platform for overall beneficial metabolic effects by forming heterodimers with RXR and modulating VDR gene expression for improved cardiovascular health. Finally, maintaining adequate levels of VD might provide a safety shield against the development of CVD and its complications (Figure 2) [83]. In addition, an identical significance was reported among seriously ill patients, among whom a low level of VD was significantly related to the severity of disease and death [100,101].

Heart failure

VDD is related to MI, post-MI, and new-onset heart failure [73,102]. Bae et al. proposed that VDD following MI in VDR knockout mice causes reduced cardiac function and survival rate, whereas VD signaling encourages cardioprotection via anti-inflammatory, anti-apoptotic, and antifibrotic mechanisms [103]. Additionally, VDD is related to enhanced inflammation and inflammatory cytokines such as TNF-α, IL-6, and IL-1β, which intercede heart failure and cardiac diseases [104]. VD supplementation decreases these cytokines during chronic heart failure [105]. IL-33 is a member of the IL-1 cytokine family. IL-33 averts cardiomyocyte apoptosis via the ST2 receptor and enhances cardiac function and survival following myocardial infarction [106]. A rise in circulating levels of soluble decoy receptors of ST2 (sST2) is related to heart failure, fibrosis, and cardiac remodeling [107]. In addition, hypoparathyroidism and low levels of VD are related to cardiomyopathy remodeling, as well as to the deterioration of heart failure [108]. Moreover, calcitriol, which is an active form of VD, plays a role in regulating cardiac function and could have a modulatory effect on ST2 [109]. Therefore, the interactions between the sST2 and VD/PTH axis that control fibrosis and inflammation within the heart may control heart failure progression. Several studies have suggested there is a strong association between low levels of VD, sST2, and heart failure and the VD/PTH (1-84) axis. It was suggested sST2 levels and a low plasma 1,25(OH)2D3/PTH (1-84) ratio are significant predictors regarding the deterioration of heart failure, hospitalization, decreased survival, and death caused by cardiac disease [110,111]. However, no association has been found between plasma levels of calcitriol, calcidiol, PTH, and the risk of heart failure development [102].

VDD is linked to heart failure, and VD supplementation could be advantageous [112]. However, several reports demonstrated no improvement or useful effect of VD in heart failure with VD supplementation; however, inconsistencies between these studies could be caused by hereditary differences in the VDR gene (e.g., Fok1)[113-115]. VD’s effects on improving the left ventricle structure and its function during HF, shown within the VINDICATE study [116], and reduced renin action with short-term VD supplementation among patients with persistent HF, suggest VD plays a beneficial role [117].

Heart failure with preserved ejection fraction (HFpEF)

This is a type of HF in which the heart pumps normally, but the heart muscles are stiff, leading to difficulties in filling the heart with enough blood. HFpEF is becoming more common, especially among older adults, and it is related to an elevated risk of hospitalization, disability, and mortality. Although the causes of HFpEF are not known, increasing evidence suggests VDD could play an important role in its progression.

VD is fat-soluble, which is important for bone health and calcium metabolism. However, it also has many nonskeletal functions, including regulating immune function, cardiovascular health, and inflammation. VD is primarily acquired through exposure to sunlight and from dietary sources (e.g., fortified foods, egg yolks, and fatty fish). However, many people have low levels of VD, especially older adults, those who have limited exposure to sunlight, and those with specific medical disorders. Numerous studies have explored the association between VD and HFpEF. Nolte et al. reported in their study that lower concentrations of 25(OH)D are related to decreased functional capability among patients with HFpEF and were considerably prognostic for an enhanced frequency of hospitalizations due to CVDs, adjusting for age, NT-proBNP, selected baseline characteristics, and comorbidities [118].

Ozdemir et al. highlighted that VD and LVEF showed a positive association among HF patients, occurring with both protective effects of VD and hypovitaminosis. Taking VD can decrease the death risk among individuals with CVD. Low levels of VD among heart failure patients are related to meager physical function [119]. Pandit et al. found an insignificant relationship between VD levels with left ventricle diastolic functioning, including the left atrial volume index [120]. A community-based, longitudinal study carried out among older adults found low levels of VD were related to an enhanced risk of HFpEF over a 10-year follow-up period [121]. Another study in which elderly patients were included was carried out in China, where lower concentrations of VD were correlated with an enhanced risk of HFpEF. The findings of this study signified that VDD may be an important risk determinant regarding HFpEF [122]. The exact mechanisms through which VDD could play a part in HFpEF development are not fully understood. However, VD may play a significant role in regulating cardiovascular function and preventing oxidative stress in addition to inflammation, which is known to contribute to the development of HFpEF [123]. Additionally, VD may improve endothelial function, which is important for maintaining proper blood flow and preventing damage to blood vessels.

Although there are growing data associating HFpEF and VDD, limited research has been carried out regarding the efficacy of VD supplementation in averting or treating HFpEF. One study found a high dose of VD supplementation improved markers of heart function among patients suffering from HFpEF [124]. However, this study was carried out on a small scale in which only patients with moderate to severe VDD were included; thus, more research is required to ascertain the optimum dosage, time duration, and timing of VD supplementation among individuals with HFpEF. Dalbeni et al. (2013) reported six months of VD supplementation considerably improves the ejection fraction among older patients suffering from VDD and heart failure [125]. Moreover, it is important to consider the potential risks and benefits regarding VD supplements in the context of an individual’s overall health and medical history. Elevated dosages of VD can be toxic, resulting in indications such as kidney damage, nausea, and vomiting. Additionally, VD supplements may react with other medicines such as BP medications and steroids. Hence, consulting with a physician is necessary before starting VD supplementation.

There is growing data highlighting that low levels of VD could boost the chance of HFpEF and that VD supplementation could have some benefits for patients with HFpEF. However, further research is required to completely understand the role played by VD in the progression and management of HFpEF, in addition to determining optimal dosage. Additionally, VD supplementation should not be viewed as a substitute for other proven treatments for HFpEF, and patients should get advice from their health-care providers to develop a comprehensive treatment plan.

Atrial fibrillation (AF)

AF is most frequent arrhythmia affecting virtually 2% of the general population and is believed to be the most significant reason for stroke, mortality, and health-care burden [32,126]. Regardless of the identification of several significant risk determinants for AF (e.g., old age, heart failure, coronary artery disease, arterial hypertension, surgery, hyperthyroidism, and valvular heart disease), an important risk factor remain currently unexplained [127]. Some research has demonstrated that low concentrations of VD are correlated with AF and could be associated with its pathogenesis, hence signifying the ability of VD to become a curative target within clinical settings [32].

The possible mechanisms by which VD could boost the risk of AF are either direct effects upon the atrium or indirect modulation of cardiovascular risk factors. Low levels of VD are related to swelling, which plays an important role in AF pathogenesis [128]. Patients with elevated values of C-reactive protein have been found to have a twofold-increased risk of AF [129]. Furthermore, low levels of VD could enhance the risk of AF caused by the renin-angiotensin-aldosterone system activation, which encourages electrical and structural atrial remodeling and controls inflammation [130]. Therefore, low levels of VD are related to elevated levels of left atrial fibrosis [131]. Eventually, low levels of VD could play a role in AF by enhancing the risk factors regarding its progression (i.e., heart failure, diabetes mellitus, CAD, and hypertension)[132]. Despite these data, the scientific evidence regarding the correlation between AF and VD is controversial [32].

The observational research has demonstrated that individuals with VDD have approximately double the risk of AF when compared to those with normal levels of VD [133,134]. The prospective research remains unsuccessful in discovering such a relationship [135,136]. Several studies have assessed the relationship between AF and VD among CVD patients or the general populace. However, in those studies that focused on the prevalence of postoperative AF, particularly following the coronary artery bypass graft, low levels of VD were consistently related to an increased risk of AF incidence [137-139]. According to this, a meta-analysis that explored the dose-response relationships determined that VDD is moderately related to an elevated risk of AF among the general populace and significantly linked to postoperative AF among individuals who had CABG surgery. Particularly, the findings of one study highlighted that the associated risk of AF was enhanced by 12%, with 56% resulting in a 10-unit reduction in VD levels among the general population and CABG patients, correspondingly, confirming a linear relationship [139]. Finally, Cerit et al. observed that VD supplementation decreased postoperative AF among patients who had CABG surgery and very low levels of VD (below 20 ng/mL) [140]. In addition, VDD seems to be related to an elevated risk of AF among patients with persistent HF [141], as well as in younger people but not the elderly [136]. It is necessary to investigate the populace and clinical settings in which the correlation between reduced levels of VD and an elevated risk of AF is more pronounced to understand whether VD supplements could eventually serve as a preventive measure for AF.

Lipids

Both observational and interventional studies have highlighted that inadequate VD concentrations are correlated with adverse serum lipid levels, whereas adequate VD concentrations are correlated with favorable lipid profiles. These findings have been confirmed by several studies [142,143]. A study conducted in 2016 on a group of Polish patients found a negative correlation among VD levels and LDL cholesterol (LDL-C), triglycerides (TGs), and total cholesterol (TC). Another study that analyzed 25(OH)D concentrations and lipid portions above 20,000 demonstrated a significant association between VDD and atherogenic lipid profile among patients [144].

Additionally, several meta-analyses have been carried out that assessed the relationship between lipid profiles, levels of VD, and VD supplementation [145-147]. A meta-analysis in 2015 that included eight randomized controlled trials (RCTs) to investigate the effect of VD supplementation on lipid profile revealed a decrease in TG levels and an increase in HDL-C and LDL-C [145]. There is a need to be cautious while interpreting these results due to the lessened number of studies and elevated heterogeneity regarding interventions and outcomes (i.e., VD supplementation dosage). A large-scale meta-analysis that evaluated the impact of VD supplementation on LDL-C, HDL-C, TC, and TG in 39 RCTs found a negative and significant association between VD supplementation and LDL-C, TC, and TG. In contrast, the use of VD supplementation elevated HDL-C levels [146]. Likewise, a meta-analysis in which seven RCTs were included reported that co-supplementation of VD and calcium (less than eight weeks of supplementation) among obese and overweight patients caused a substantial fall in LDL-C, TG, and TC and an increase in HDL-C [147]. Low levels of VD have been shown in polycystic ovary syndrome, particularly among patients with obesity and a waist-to-hip ratio greater than 0.85 [148]. Moreover, VDD can potentially increase the susceptibility to endocrine-metabolic disorder [149]. One meta-analysis of 11 RCTs in which 483 Polycystic Ovary Syndrome patients were included to evaluate the impact of VD supplementation versus placebo revealed the patients treated with VD supplementation demonstrated a decrease in TC and insulin resistance versus patients treated with a placebo. However, VD supplementation did not boost TG and HDL-C levels among PCOS patients [150]. Among children, VD supplementation for the maintenance of serum 25(OH)D within an optimal range is linked to an insignificant risk of T1DM development and helps control the disease action [151].

VD supplementation and cardiovascular health

A recent study was carried out to evaluate the cardiovascular results among patients using vitamin D3 or omega-3 supplements in the general population. During the study, 25,871 patients from the United States were enrolled. Patients included men above 50 years and women above 55 years of age. The participants took a placebo or daily dose of 2,000 IU of VD. The major endpoints were reduced in stroke, MI, and mortality from cardiovascular reasons followed by an average of 5.3 years. The findings of the study found insignificant advantages in cardiovascular results. There was no significant decrease observed in cardiovascular incidents among patients who received VD when compared with patients who received a placebo [152]. These outcomes were found to be consistent with the Women’s Health Initiative Calcium & VD Trial. The study found there were no cardiovascular advantages of daily intake of VD supplements [153].

The DIMENSION study evaluated the possible effect of 16-week cholecalciferol supplements on endothelial activity among diabetic patients. Particularly, ultimate enhancements were evaluated within vascular biological markers, as well as the reactive hyperemia index. The levels of VD were considerably raised in the treatment arm. However, one multivariate regression analysis showed no impact on endothelial function [154]. Another study that evaluated the probable VD protective action on the indicator of heart lesions demonstrated that cholecalciferol, dispensed before a percutaneous coronary technique, did not show any MACE alteration when compared with the control [155]. A study was carried out in a primary health-care facility among healthy participants to assess the outcomes of a one-year daily supplement of cholecalciferol on the risk of disease and biochemical indicators. The outcomes of this study were not promising. The administration of VD improved serum 25(OH) levels; however, no substantial enhancements were observed concerning cardiovascular risk factors, arterial rigidity, blood lipids, and BP [156]. A D-Health Trial was carried out among 21,000 respondents to assess VD supplementation effectiveness for the preclusion of carcinoma and death. Specifically, this study was performed to assess the impact of placebos or monthly oral doses of cholecalciferol over five years, followed by another five years of passive surveillance using health records and mortality databases. However, the outcomes of the study remained unsuccessful in elucidating whether VD supplements employed any defensive actions on the risks of carcinoma and mortality. It was concluded during the study that data acquired from the observational studies did not help support VD utilization among healthy individuals as a defense agent [157]. Furthermore, the cholecalciferol supplement did not decrease the risk of CVD [158].

Another study was conducted to explore whether the use of a cholecalciferol supplement for 12 weeks daily may be beneficial among healthy people to reduce heart rate, BP, and other CVD risk indicators. This therapy enhanced 25(OH)D serum levels but did not improve CVD risk [159]. Another study assessed the relationship between CVD and serum 25(OH)D concentrations but observed no significant association; however, low levels of VD were associated with a 44% rise in CVD risk, with an elevated death rate associated with CVD [160]. Another meta-analysis was carried out in which 21 RCTs were included to assess the cardiovascular advantages of VD supplementation for one year, irrespective of calcium supplement. The results showed the main endpoint was an amalgamation of MACEs, whereas the secondary endpoint comprised the final alterations involving stroke, cerebrovascular accidents, MI cardiovascular death, and all-cause death. The outcomes of this meta-analysis indicated VD supplementation did not produce any considerable alterations in MACE, solo cardiovascular endpoints (stroke, MI, cardiovascular death), or all-cause death [161]. The outcomes of these studies are in line with the evidence that VD supplementation does not provide pertinent benefits regarding cardiovascular health. Incidences of VD resulting in toxic effects are very uncommon because VD toxicity could be encouraged due to elevated doses. VD toxicity may result in hypercalcemia, which can lead to sustained cardiac arrhythmias caused by a reduced QT interval.

VD toxicity

Signs and Symptoms

VD toxicity is attributed to hypercalciuria or hypercalcemia, along with typical signs (abdominal pain, lethargy, constipation, anorexia, nocturia, and polyuria) directly caused by these anomalies. In several cases, the symptomatology associated with hypercalciuria or hypercalcemia is insignificant. Although, as identified in case reports and case series, the long-term persistence of insignificant biochemical anomalies or development to acute electrolyte commotions can cause more grave issues, such as renal dysfunction, dehydration, and nephrocalcinosis [162].

Toxic Threshold Levels

At present, no universally accepted 25OHD level serves as a threshold for the development of risk. However, this threshold is typically between the values of 250 and 750 nmol/L. Despite the lack of evidence indicating that children exhibit biochemical abnormalities or signs with 25OHD values at or somewhat >250 nmol/L, current pediatric clinical studies involving high dosages of VD have focused on this threshold [163]. Utilizing this threshold for dosing studies is suitable because levels of more than this are found to be supraphysiological, meaning they cannot be attained with healthy diets or excessive sun exposure. Additionally, no evidence is available regarding any benefits associated with 25OHD dosages of more than 200 nmol/L [164].

Risk Factors for VD-Related Toxicity

Regardless of the public and clinical concerns about the toxicity of VD, it is an uncommon event that mostly takes place in terms of hereditary vulnerability or incorrect intake of elevated dosages of VD [162]. Apprehension regarding the safety of consuming greater than 4,000 IU of VD daily can be traced back to the 1950s. During this time, there was a surge in cases of idiopathic infantile hypercalcemia, which occurred at the same time as the widespread adoption of a daily VD intake of approximately 4,000 IU/day [162]. This minor epidemic caused a reduction in the daily recommended consumption to avoid rickets, as well as hypocalcemic seizures (400 IU daily). It was debated that many, possibly all, of the idiopathic infantile hypercalcemia cases were caused by rare hereditary disorders (<1:10,000) that enhance the vulnerability to VD toxicity [165]. Among these, patients who have Williams syndrome may exhibit heart defects as part of their range of indications, and it would be advisable to evade excessive VD ingestion in this particular subgroup [166].

A significant amount of low-level evidence indicates that consuming elevated dosages of VD, resulting in a shorter-term aggregated ingestion of 600,000 IU or more, could be excessive and may cause hypercalciuria, hypercalcemia, and finally nephrocalcinosis. In addition, this circumstantial evidence is corroborated by a prospective clinical trial among pediatric patients, which showed significant rates of hypercalcemia among healthy children who were intermittently given high dosages of 600,000 IU, mostly in a repeated manner [162]. In the majority of cases, when a diagnosis is made and the source of unnecessary VD is stopped, there is a gradual decrease in BP levels below noxious levels. This typically causes resolution of indications, as well as biochemical anomalies. Among certain cases, even after discontinuation of VD, nephrocalcinosis was found to persist. A literature review of nephrocalcinosis highlights that most of the cases related to VD have been observed among children diagnosed with an uncommon hereditary disorder known as VD-resistant rickets. In these cases, the condition may be linked to simultaneous phosphate ingestion [167,168]. Again, an evaluation of case reports and series indicates that, among healthy kids, the occurrence of nephrocalcinosis is only seen with intentional/unintentional cumulative VD ingestion of more than 600,000 IU [167,169,170]. An analysis of interventional studies in pediatric patients, focusing on VD supplementation, found that two studies examined daily high-dose VD at levels approaching but not exceeding the upper limit recommended by the Institute of Medicine, whereas four studies employed megadoses ranging from 100,000 to 150,000 IU. None of the studies reported raised urinary calcium excretion/hypercalciuria [171,172]. Based on these results, it would be advisable to refrain from administering toral VD dosages at or near 600,000 IU [162].

VD and COVID-19

VD administration may decrease the prevalence of viral respiratory tract infections, mostly among cases in which a deficiency of this nutrient is present [12,173]. VD is a vital component of the immune system, functioning as an immunomodulatory hormone that possesses anti-inflammatory and antimicrobial properties. This observation may account for the protective and beneficial effects associated with VD in averting severe acute respiratory syndrome coronavirus-2 (SARS-CoV-2) infectivity, decreasing viral replication, promoting rapid viral clearance, and limiting its spread. Similarly, VDD harms the circulatory system and is related to CVD. COVID-19 severity is found to be worse among persons who have a history of CVD [174]. About 25% of patients with the disease have been found to experience myocardial injury, with several developing important cardiac symptoms such as biventricular HF, arrhythmias, and, infrequently, cardiogenic shock and mortality [175,176].

VD exhibits several beneficial effects. It controls the adaptive immune system, possesses anti-inflammatory properties, encourages the expression of several molecules that participate in the antioxidant defense system, reduces cellular oxidation and oxidative stress, and demonstrates vasoprotective effects [177]. In addition, VD plays an important role in promoting the expression of various molecules that participate in the antioxidant defense system. It also modifies immune function, facilitates viral elimination, and lowers inflammatory responses by decreasing the production of inflammatory cytokines such as interleukin-6, -8, -12, and -17. VD levels are related to decreased levels of interleukin-6, which plays a part in the cytokine storm found among critically ill individuals and is related to a poorer COVID-19 prognosis. A small-scale study was carried out by Kox et al. to compare the levels of cytokines among critically ill individuals with COVID-19 and individuals with other critical illnesses. The findings of the study highlighted that plasma levels of TNF, interleukin-6, and interleukin-8 were considerably lower among COVID-19 patients than among septic shock patients with acute respiratory distress syndrome, which elucidates a lower acuteness of disease despite acute lung injury. Hence, the findings of the study recommend that COVID-19 severity may not be caused by a cytokine storm [178]. Likewise, VD boosts the angiotensin-converting enzyme-2 level in the lungs, an important receptor for severe respiratory syndrome [179,180].

One study investigated the relationship between VD concentrations and the prevalence of COVID-19 [180]. The individuals with deficient VD levels during administration of a COVID-19 test showed a significantly elevated risk of testing positive than those within a normal range. In addition, individuals who received VD supplementation before the epidemic did not exhibit a higher risk of contracting COVID-19 when compared with those with normal concentrations of VD and not supplemented. This suggests VD supplementation may have a protective effect against COVID-19. Collectively, these outcomes signify that administering high-dose VD treatment to rapidly replenish circulating VD concentrations may lower the risk of COVID-19 severity, as well as mortality [181]. Individuals with persistent conditions (e.g., elderly people, smokers, obese individuals, those with type-2 diabetes mellitus, and African Americans), have lower levels of VD, which may lead to more severe SARS-CoV-2 infectivity [182]. VDD may be an important risk determinant regarding sternness of COVID-19 infectivity. Hence, individuals at risk of VDD may benefit from VD supplementation. This approach is affordable, accessible, and considered safe. However, at present, there is insufficient scientific evidence encouraging the clinical routine usage of VD among patients with COVID-19 [183].

A compelling discussion has arisen regarding the effectiveness of administering high-dose VD supplementation to mitigate the risk of COVID-19 infectivity and severity. Thus, further clinical studies are needed to obtain stronger evidence about the impact of VD in lowering the prevalence of hospitalization and mortality. Additionally, there is a need to determine whether VD supplementation decreases the likelihood of SARS-CoV-2 virus infection [12].

VD in noncardiac surgery

More and more patients with various clinical situations have been found to have VDD, which is why various disciplines of medicine encounter such patients [184]. Surgeons dealing with patients undergoing noncardiac surgery and anesthesiologists dealing with patients in intensive care settings and long-term pain clinics have also detected VDD in their patients. VDD might impact the outcomes of such patients. There has been a lot of discussion on these issues recently, and it has been observed that low levels of VD are positively correlated with extended duration of stay and readmission to ICU within 90 days, as well as significantly contributing to mortality [185].

The outcomes of one meta-analysis highlighted that severe VDD could be independently related to enhanced mortality risk among elderly patients with sepsis [186]. One study indicated that, among patients with sepsis admitted to a health facility, low levels of VD were related to enhanced mortality [187], whereas the results of another study reported that, for patients who experienced noncardiac surgical treatment, optimal VD concentration decreased the risk of death [188]. These findings could be elucidated by the significant immunomodulatory function of VD, as well as its association with persistent diseases [189].

Anesthesia consideration

VD is a vital fat-soluble vitamin that plays a crucial role in the maintenance of bone health and calcium homeostasis. In recent years, VDD has been linked to a variety of unfavorable health outcomes such as diabetes mellitus, CVD, and carcinoma. In the context of cardiac surgery, VDD can have several consequences for the anesthesiologist, including impaired immune function, increased risk of post-op complications, impaired bone health, and increased risk of CVD [80].

Impaired Immune Function

VD plays an important role in controlling the immune system. VDD has been related to an enhanced risk of contagions and impaired wound healing, which can be particularly problematic in the context of cardiac surgical treatment. Patients experiencing cardiac surgical treatment are already at an enhanced risk of infection caused by the invasive nature of the surgical technique and the need for prolonged hospitalization. VDD may further increase this risk by impairing immune cell function and decreasing the production of antimicrobial peptides [190].

Anesthesiologists may need to take extra precautions to prevent infections in patients with VDD. This may include implementing strict infection control protocols, such as hand hygiene and environmental cleaning, and ensuring patients receive appropriate prophylactic antibiotics. Anesthesiologists may also need to monitor wound healing more closely among patients with VDD, as impaired wound healing can lead to surgical site infections and delayed recovery [191].

Increased Risk of Postoperative Complications

VDD has been associated with an enhanced risk of postoperative complications, such as arrhythmias, infections, and acute kidney injury (AKI). These complications can be particularly problematic in the context of cardiac surgery, as they can lead to prolonged hospitalization, increased health-care costs, and decreased quality of life for the patient [192].

Arrhythmias are a common complication of cardiac surgery, with up to 30% of patients experiencing some form of arrhythmia during or after surgery. VDD has been associated with an enhanced risk of arrhythmias, comprising AF and ventricular tachycardia. Anesthesiologists may need to monitor patients with VDD more closely for signs of arrhythmias and adjust their treatment plans as necessary. This may include administering antiarrhythmic medications, such as beta-blockers or amiodarone, and ensuring electrolyte imbalances, such as hypokalemia and hypomagnesemia, are corrected [56].

Infections are another common complication of cardiac surgery, with up to 10% of patients experiencing some form of infection after surgery. VDD has been associated with an enhanced risk of infections, such as surgical site infections and pneumonia. Anesthesiologists may need to be particularly vigilant in monitoring patients with VDD for signs of infection and adjusting their treatment plans as necessary. This may include administering appropriate antibiotics, ensuring adequate hydration, and optimizing nutrition [193].

AKI is a serious complication from cardiac surgical treatment, with up to 30% of patients experiencing some form of AKI after surgery. VDD has been associated with an enhanced risk of AKI, possibly caused by the role played by VD in regulating renal function and BP. Anesthesiologists may need to monitor patients with VDD more closely for signs of AKI and adjust their treatment plans accordingly. This may include optimizing hemodynamics, ensuring adequate hydration, and avoiding nephrotoxic medications [194].

Impaired Bone Health

VD is important for bone health, and VDD can cause a range of bone-related problems, including osteoporosis and fractures. In the context of cardiac surgery, patients may be immobilized for extended periods, which could exacerbate any underlying bone problems. Anesthesiologists may need to take steps to prevent or manage these complications, such as ensuring adequate pain relief, encouraging early mobilization, and providing supplements containing calcium and VD, as needed [195].

VDD can result in reduced calcium absorption and elevated parathyroid hormone (PTH) levels, leading to bone resorption and decreased bone density. This can increase the risk of fractures, which can be especially problematic for patients who are immobilized after cardiac surgery. Anesthesiologists may need to pay particular attention to bone health in patients with preexisting bone conditions or those at risk for bone-related complications [80].

In addition to VD and calcium supplementation, anesthesiologists may also consider other strategies to promote bone health, such as optimizing nutrition, encouraging weight-bearing activities, and monitoring bone density using techniques such as dual-energy X-ray absorptiometry scans. By taking a proactive approach to managing bone health among patients with VDD, anesthesiologists can help minimize the risk of complications and improve outcomes after cardiac surgery [196].

Increased Risk of CVD

VDD has been associated with an enhanced risk of CVDs, comprising hypertension, HF, and myocardial infarction. Cardiac surgery patients are already at an enhanced risk of cardiovascular complications, and VDD may further exacerbate this risk. Anesthesiologists may need to take extra precautions for managing patients with VDD to minimize their risk of cardiovascular complications during and after surgery. This may include optimizing BP control, ensuring adequate fluid management, and monitoring for signs of myocardial ischemia [197].

In addition to these specific consequences, VDD may have broader implications for the overall perioperative care of cardiac surgery patients. For example, VDD has been associated with an enhanced risk of cognitive deterioration and delirium, which could impact postoperative recovery and outcomes. Anesthesiologists may need to consider the potential effect of VDD on these outcomes and take steps to mitigate any negative effects.

Overall, VDD can have significant consequences for anesthesiologists in the context of cardiac surgery. Anesthesiologists should be aware of the potential risks and take steps to prevent or manage any complications that may arise. This may include screening patients for VDD before surgery, optimizing nutrition and supplementation, and closely monitoring patients for signs of infection, arrhythmias, AKI, bone-related problems, and CVD. By proactively addressing VDD, anesthesiologists can help improve outcomes and optimize the overall perioperative care of cardiac surgery patients.

## Conclusions

Overall, the association between VD and cardiovascular risk is complex and still not fully understood. Although observational studies have suggested low concentrations of VD may be related to enhanced risk of CVD, the evidence from RCTs of VD supplementation for CVD prevention or management is mixed.

The potential mechanisms through which VD may influence cardiovascular risk include its effects on inflammation, oxidative stress, BP, glucose metabolism, and lipid metabolism. However, the optimal dosage and duration of VD supplementation for cardiovascular health are still unclear, and more studies are required to clarify its role and identify the populations that may benefit most from supplementation.

Meanwhile, it is critical for individuals to maintain a healthy lifestyle that includes regular physical activity, a balanced and varied diet, and no smoking, as these are well-established factors to reduce cardiovascular risk. For individuals with low levels of VD or other risk factors regarding CVD, it is important to talk to their healthcare provider regarding the potential benefits and risks of VD supplementation and to follow their recommended management plan.

## References

[1] Kheiri B, Abdalla A, Osman M, Ahmed S, Hassan M, Bachuwa G (2018). Vitamin D deficiency and risk of cardiovascular diseases: a narrative review. Clin Hypertens.

[2] Artaza JN, Contreras S, Garcia LA (2011). Vitamin D and cardiovascular disease: potential role in health disparities. J Health Care Poor Underserved.

[3] Zhang R, Naughton DP (2010). Vitamin D in health and disease: current perspectives. Nutr J.

[4] Demer LL, Hsu JJ, Tintut Y (2018). Steroid hormone vitamin D: implications for cardiovascular disease. Circ Res.

[5] DeLuca HF (2004). Overview of general physiologic features and functions of vitamin D. Am J Clin Nutr.

[6] Kessi-Pérez EI, González A, Palacios JL, Martínez C (2022). Yeast as a biological platform for vitamin D production: a promising alternative to help reduce vitamin D deficiency in humans. Yeast.

[7] Guo J, Lovegrove JA, Givens DI (2018). 25(OH)D3-enriched or fortified foods are more efficient at tackling inadequate vitamin D status than vitamin D3. Proc Nutr Soc.

[8] Jorge AJL, Cordeiro JR, Rosa MLG, Bianchi DBC (2018). Vitamin D deficiency and cardiovascular diseases. Int J Cardiovasc Sci.

[9] Zittermann A, Pilz S (2019). Vitamin D and cardiovascular disease: an update. Anticancer Res.

[10] Wang TJ (2016). Vitamin D and cardiovascular disease. Annu Rev Med.

[11] Hagău AC, Pușcaș A, Togănel R, Muntean I (2023). Is hypovitaminosis D a risk factor for heart failure?. Life (Basel).

[12] de la Guía-Galipienso F, Martínez-Ferran M, Vallecillo N, Lavie CJ, Sanchis-Gomar F, Pareja-Galeano H (2021). Vitamin D and cardiovascular health. Clin Nutr.

[13] Charoenngam N, Shirvani A, Holick MF (2019). Vitamin D for skeletal and non-skeletal health: what we should know. J Clin Orthop Trauma.

[14] Pludowski P, Holick MF, Grant WB (2018). Vitamin D supplementation guidelines. J Steroid Biochem Mol Biol.

[15] Holick MF (2006). Resurrection of vitamin D deficiency and rickets. J Clin Invest.

[16] Bikle DD (2014). Vitamin D metabolism, mechanism of action, and clinical applications. Chem Biol.

[17] Cooke NE, Haddad JG (1989). Vitamin D binding protein (Gc-globulin). Endocr Rev.

[18] Valero Zanuy MÁ, Hawkins Carranza F (2007). Metabolism, endogenous and exogenous sources of vitamin D. Rev Esp Enfermedades Metab Oseas.

[19] Lehmann B, Tiebel O, Meurer M (1999). Expression of vitamin D3 25-hydroxylase (CYP27) mRNA after induction by vitamin D3 or UVB radiation in keratinocytes of human skin equivalents: a preliminary study. Arch Dermatol Res.

[20] Bikle DD, Nemanic MK, Whitney JO, Elias PW (1986). Neonatal human foreskin keratinocytes produce 1,25-dihydroxyvitamin D3. Biochemistry.

[21] Vantieghem K, Kissmeyer AM, De Haes P, Bouillon R, Segaert S (2006). UVB-induced production of 1,25-dihydroxyvitamin D3 and vitamin D activity in human keratinocytes pretreated with a sterol delta7-reductase inhibitor. J Cell Biochem.

[22] Segaert S, Simonart T (2008). The epidermal vitamin D system and innate immunity: some more light shed on this unique photoendocrine system?. Dermatology.

[23] Bikle DD (2004). Vitamin D regulated keratinocyte differentiation. J Cell Biochem.

[24] Chowdhury R, Kunutsor S, Vitezova A (2014). Vitamin D and risk of cause specific death: systematic review and meta-analysis of observational cohort and randomised intervention studies. BMJ.

[25] Bikle D, Christakos S (2020). New aspects of vitamin D metabolism and action - addressing the skin as source and target. Nat Rev Endocrinol.

[26] Maretzke F, Bechthold A, Egert S (2020). Role of vitamin D in preventing and treating selected extraskeletal diseases—an umbrella review. Nutrients.

[27] Siotto M, Santoro M, Aprile I (2020). Vitamin D and rehabilitation after stroke: status of art. Appl Sci.

[28] Ross CA, Taylor CL, Yaktime AL (2011). Dietary Reference Intakes for Calcium and Vitamin; Committee to Review Dietary Reference Intakes for Vitamin D and Calcium.

[29] Holick MF, Binkley NC, Bischoff-Ferrari HA (2011). Evaluation, treatment, and prevention of vitamin D deficiency: an Endocrine Society clinical practice guideline. J Clin Endocrinol Metab.

[30] Romagnoli E, Carnevale V, Biondi P, Minisola S (2014). Vitamin D supplementation: when and how?. J Endocrinol Invest.

[31] Zittermann A, Trummer C, Theiler-Schwetz V, Lerchbaum E, März W, Pilz S (2021). Vitamin D and cardiovascular disease: an updated narrative review. Int J Mol Sci.

[32] Cosentino N, Campodonico J, Milazzo V, De Metrio M, Brambilla M, Camera M, Marenzi G (2021). Vitamin D and cardiovascular disease: current evidence and future perspectives. Nutrients.

[33] Khammissa RA, Fourie J, Motswaledi MH, Ballyram R, Lemmer J, Feller L (2018). The biological activities of vitamin D and its receptor in relation to calcium and bone homeostasis, cancer, immune and cardiovascular systems, skin biology, and oral health. Biomed Res Int.

[34] Hiemstra TF, Lim K, Thadhani R, Manson JE (2019). Vitamin D and atherosclerotic cardiovascular disease. J Clin Endocrinol Metab.

[35] Freundlich M, Li YC, Quiroz Y (2013). Paricalcitol downregulates myocardial renin-angiotensin and fibroblast growth factor expression and attenuates cardiac hypertrophy in uremic rats. Am J Hypertens.

[36] Cui C, Xu P, Li G (2019). Vitamin D receptor activation regulates microglia polarization and oxidative stress in spontaneously hypertensive rats and angiotensin II-exposed microglial cells: role of renin-angiotensin system. Redox Biol.

[37] Oh J, Matkovich SJ, Riek AE (2020). Macrophage secretion of miR-106b-5p causes renin-dependent hypertension. Nat Commun.

[38] Polly P, Tan TC (2014). The role of vitamin D in skeletal and cardiac muscle function. Front Physiol.

[39] Zittermann A (2018). Vitamin D status, supplementation and cardiovascular disease. Anticancer Res.

[40] Nakashima A, Yokoyama K, Yokoo T, Urashima M (2016). Role of vitamin D in diabetes mellitus and chronic kidney disease. World J Diabetes.

[41] Roffe-Vazquez DN, Huerta-Delgado AS, Castillo EC (2019). Correlation of Vitamin D with inflammatory cytokines, atherosclerotic parameters, and lifestyle factors in the setting of heart failure: a 12-month follow-up study. Int J Mol Sci.

[42] Latic N, Erben RG (2020). Vitamin D and cardiovascular disease, with emphasis on hypertension, atherosclerosis, and heart failure. Int J Mol Sci.

[43] Condoleo V, Pelaia C, Armentaro G (2021). Role of vitamin D in cardiovascular diseases. Endocrines.

[44] Adams JS, Ren S, Liu PT (2009). Vitamin d-directed rheostatic regulation of monocyte antibacterial responses. J Immunol.

[45] Dickie LJ, Church LD, Coulthard LR (2010). Vitamin D3 down-regulates intracellular Toll-like receptor 9 expression and Toll-like receptor 9-induced IL-6 production in human monocytes. Rheumatology.

[46] Chen S, Sims GP, Chen XX, Gu YY, Chen S, Lipsky PE (2007). Modulatory effects of 1,25-dihydroxyvitamin D3 on human B cell differentiation. J Immunol.

[47] Boonstra A, Barrat FJ, Crain C, Heath VL, Savelkoul HF, O'Garra A (2001). 1alpha,25-Dihydroxyvitamin d3 has a direct effect on naive CD4(+) T cells to enhance the development of Th2 cells. J Immunol.

[48] Oh J, Weng S, Felton SK (2009). 1,25(OH)2 vitamin d inhibits foam cell formation and suppresses macrophage cholesterol uptake in patients with type 2 diabetes mellitus. Circulation.

[49] Yin K, You Y, Swier V, Tang L, Radwan MM, Pandya AN, Agrawal DK (2015). Vitamin D protects against atherosclerosis via regulation of cholesterol efflux and macrophage polarization in hypercholesterolemic swine. Arterioscler Thromb Vasc Biol.

[50] Chen S, Swier VJ, Boosani CS, Radwan MM, Agrawal DK (2016). Vitamin D deficiency accelerates coronary artery disease progression in swine. Arterioscler Thromb Vasc Biol.

[51] Kasuga H, Hosogane N, Matsuoka K, Mori I, Sakura Y, Shimakawa Shimakawa (2002). Characterization of transgenic rats constitutively expressing vitamin D-24-hydroxylase gene. Biochem Biophys Res Commun.

[52] Carbone F, Satta N, Burger F (2016). Vitamin D receptor is expressed within human carotid plaques and correlates with pro-inflammatory M1 macrophages. Vascul Pharmacol.

[53] Neeland IJ, Poirier P, Després JP (2018). Cardiovascular and metabolic heterogeneity of obesity: clinical challenges and implications for management. Circulation.

[54] Poole KE, Loveridge N, Barker PJ, Halsall DJ, Rose C, Reeve J, Warburton EA (2006). Reduced vitamin D in acute stroke. Stroke.

[55] Liu AJ, Guo JM, Xia W, Su DF (2010). New strategies for the prevention of stroke. Clin Exp Pharmacol Physiol.

[56] Pilz S, Tomaschitz A, Drechsler C, Zittermann A, Dekker JM, März W (2011). Vitamin D supplementation: a promising approach for the prevention and treatment of strokes. Curr Drug Targets.

[57] Pittas AG, Lau J, Hu FB, Dawson-Hughes B (2007). The role of vitamin D and calcium in type 2 diabetes. A systematic review and meta-analysis. J Clin Endocrinol Metab.

[58] Muscogiuri G, Annweiler C, Duval G (2017). Vitamin D and cardiovascular disease: from atherosclerosis to myocardial infarction and stroke. Int J Cardiol.

[59] Brøndum-Jacobsen P, Nordestgaard BG, Schnohr P, Benn M (2013). 25-hydroxyvitamin D and symptomatic ischemic stroke: an original study and meta-analysis. Ann Neurol.

[60] Zhou R, Wang M, Huang H, Li W, Hu Y, Wu T (2018). Lower vitamin D status is associated with an increased risk of ischemic stroke: a systematic review and meta-analysis. Nutrients.

[61] Judd SE, Morgan CJ, Panwar B (2016). Vitamin D deficiency and incident stroke risk in community-living black and white adults. Int J Stroke.

[62] Pilz S, Dobnig H, Fischer JE (2008). Low vitamin D levels predict stroke in patients referred to coronary angiography. Stroke.

[63] Makariou SE, Michel P, Tzoufi MS, Challa A, Milionis HJ (2014). Vitamin D and stroke: promise for prevention and better outcome. Curr Vasc Pharmacol.

[64] Manson JE, Cook NR, Lee IM, Christen W, Bassuk SS, Mora S (2019). Vitamin D supplements and prevention of cancer and cardiovascular disease. N Engl J Med.

[65] Pittas AG, Dawson-Hughes B (2010). Vitamin D and diabetes. J Steroid Biochem Mol Biol.

[66] Mohr SB, Garland CF, Gorham ED, Garland FC (2008). The association between ultraviolet B irradiance, vitamin D status and incidence rates of type 1 diabetes in 51 regions worldwide. Diabetologia.

[67] Zipitis CS, Akobeng AK (2008). Vitamin D supplementation in early childhood and risk of type 1 diabetes: a systematic review and meta-analysis. Arch Dis Child.

[68] Tenconi MT, Devoti G, Comelli M, Pinon M, Capocchiano A, Calcaterra V, Pretti G (2007). Major childhood infectious diseases and other determinants associated with type 1 diabetes: a case-control study. Acta Diabetol.

[69] Liu E, Meigs JB, Pittas AG, McKeown NM, Economos CD, Booth SL, Jacques PF (2009). Plasma 25-hydroxyvitamin d is associated with markers of the insulin resistant phenotype in nondiabetic adults. J Nutr.

[70] Ock SY, Ha KH, Kim BK (2016). Serum 25-hydroxy vitamin D concentration is independently inversely associated with insulin resistance in the healthy, non-obese Korean population. Diabetes Metab J.

[71] Nardin M, Verdoia M, Schaffer A, Barbieri L, Marino P, De Luca G (2016). Vitamin D status, diabetes mellitus and coronary artery disease in patients undergoing coronary angiography. Atherosclerosis.

[72] Schafer AL, Napoli N, Lui L, Schwartz AV, Black DM (2014). Serum 25-hydroxyvitamin D concentration does not independently predict incident diabetes in older women. Diabet Med.

[73] Rai V, Agrawal DK (2017). Role of vitamin D in cardiovascular diseases. Endocrinol Metab Clin North Am.

[74] Chen S, Sun Y, Agrawal DK (2015). Vitamin D deficiency and essential hypertension. J Am Soc Hypertens.

[75] Yilmaz S, Sen F, Ozeke O, Temizhan A, Topaloglu S, Aras D, Aydogdu S (2015). The relationship between vitamin D levels and nondipper hypertension. Blood Press Monit.

[76] Li YC, Qiao G, Uskokovic M, Xiang W, Zheng W, Kong J (2004). Vitamin D: a negative endocrine regulator of the renin-angiotensin system and blood pressure. J Steroid Biochem Mol Biol.

[77] Zagami RM, Di Pino A, Urbano F, Piro S, Purrello F, Rabuazzo AM (2015). Low circulating vitamin D levels are associated with increased arterial stiffness in prediabetic subjects identified according to HbA1c. Atherosclerosis.

[78] Kang JY, Kim MK, Jung S, Shin J, Choi BY (2016). The cross-sectional relationships of dietary and serum vitamin D with cardiometabolic risk factors: Metabolic components, subclinical atherosclerosis, and arterial stiffness. Nutrition.

[79] Kassi E, Adamopoulos C, Basdra EK, Papavassiliou AG (2013). Role of vitamin D in atherosclerosis. Circulation.

[80] Holick MF (2007). Vitamin D deficiency. N Engl J Med.

[81] Oruc CU, Akpinar YE, Amikishiyev S, Uzum AK, Salmaslioglu A, Gurdol F, Omer B (2017). Hypovitaminosis D is associated with endothelial dysfunction in patients with metabolic syndrome. Curr Vasc Pharmacol.

[82] Ni W, Watts SW, Ng M, Chen S, Glenn DJ, Gardner DG (2014). Elimination of vitamin D receptor in vascular endothelial cells alters vascular function. Hypertension.

[83] Sarhan N, Essam Abou Warda A, Alsahali S, Alanazi AS (2023). Dietary reference intakes for calcium and vitamin; committee to review Dietary Reference Intakes for vitamin D and calcium. Pharmaceuticals (Basel).

[84] De Metrio M, Milazzo V, Rubino M (2015). Vitamin D plasma levels and in-hospital and 1-year outcomes in acute coronary syndromes: a prospective study. Medicine (Baltimore).

[85] Giovannucci E, Liu Y, Hollis BW, Rimm EB (2008). 25-hydroxyvitamin D and risk of myocardial infarction in men: a prospective study. Arch Intern Med.

[86] Lee JH, Gadi R, Spertus JA, Tang F, O'Keefe JH (2011). Prevalence of vitamin D deficiency in patients with acute myocardial infarction. Am J Cardiol.

[87] Correia LC, Sodré F, Garcia G (2013). Relation of severe deficiency of vitamin D to cardiovascular mortality during acute coronary syndromes. Am J Cardiol.

[88] Bahrami LS, Ranjbar G, Norouzy A, Arabi SM (2020). Vitamin D supplementation effects on the clinical outcomes of patients with coronary artery disease: a systematic review and meta-analysis. Sci Rep.

[89] Bischoff-Ferrari HA, Vellas B, Rizzoli R (2020). Effect of vitamin D supplementation, omega-3 fatty acid supplementation, or a strength-training exercise program on clinical outcomes in older adults: the DO-HEALTH randomized clinical trial. JAMA.

[90] LeBoff MS, Murata EM, Cook NR (2020). Vitamin D and omega-3 trial (VITAL): effects of vitamin D supplements on risk of falls in the US population. J Clin Endocrinol Metab.

[91] Lund B, Badskjaer J, Lund B, Soerensen OH (1978). Vitamin D and ischaemic heart disease. Horm Metab Res.

[92] Scragg R, Jackson R, Holdaway IM, Lim T, Beaglehole R (1990). Myocardial infarction is inversely associated with plasma 25-hydroxyvitamin D3 levels: a community-based study. Int J Epidemiol.

[93] Wang TJ, Pencina MJ, Booth SL (2008). Vitamin D deficiency and risk of cardiovascular disease. Circulation.

[94] Wang L, Song Y, Manson JE (2012). Circulating 25-hydroxy-vitamin D and risk of cardiovascular disease: a meta-analysis of prospective studies. Circ Cardiovasc Qual Outcomes.

[95] Ng LL, Sandhu JK, Squire IB, Davies JE, Jones DJ (2013). Vitamin D and prognosis in acute myocardial infarction. Int J Cardiol.

[96] Aleksova A, Belfiore R, Carriere C, Kassem S, La Carrubba S, Barbati G, Sinagra G (2015). Vitamin D deficiency in patients with acute myocardial infarction: an Italian single-center study. Int J Vitam Nutr Res.

[97] Khalili H, Talasaz AH, Salarifar M (2012). Serum vitamin D concentration status and its correlation with early biomarkers of remodeling following acute myocardial infarction. Clin Res Cardiol.

[98] De Metrio M, Milazzo V, Marenzi G (2012). Serum vitamin D concentration status and its correlation with early biomarkers of remodeling following acute myocardial infarction. Clin Res Cardiol.

[99] Pilz S, März W, Wellnitz B (2008). Association of vitamin D deficiency with heart failure and sudden cardiac death in a large cross-sectional study of patients referred for coronary angiography. J Clin Endocrinol Metab.

[100] Perron RM, Lee P (2013). Efficacy of high-dose vitamin D supplementation in the critically ill patients. Inflamm Allergy Drug Targets.

[101] Moromizato T, Litonjua AA, Braun AB, Gibbons FK, Giovannucci E, Christopher KB (2014). Association of low serum 25-hydroxyvitamin D levels and sepsis in the critically ill. Crit Care Med.

[102] Meems LM, Brouwers FP, Joosten MM (2016). Plasma calcidiol, calcitriol, and parathyroid hormone and risk of new onset heart failure in a population-based cohort study. ESC Heart Fail.

[103] Bae S, Singh SS, Yu H, Lee JY, Cho BR, Kang PM (2013). Vitamin D signaling pathway plays an important role in the development of heart failure after myocardial infarction. J Appl Physiol (1985).

[104] Gullestad L, Ueland T, Vinge LE, Finsen A, Yndestad A, Aukrust P (2012). Inflammatory cytokines in heart failure: mediators and markers. Cardiology.

[105] Schleithoff SS, Zittermann A, Tenderich G, Berthold HK, Stehle P, Koerfer R (2006). Vitamin D supplementation improves cytokine profiles in patients with congestive heart failure: a double-blind, randomized, placebo-controlled trial. Am J Clin Nutr.

[106] Seki K, Sanada S, Kudinova AY, Steinhauser ML, Handa V, Gannon J, Lee RT (2009). Interleukin-33 prevents apoptosis and improves survival after experimental myocardial infarction through ST2 signaling. Circ Heart Fail.

[107] Yao HC, Li XY, Han QF (2015). Elevated serum soluble ST2 levels may predict the fatal outcomes in patients with chronic heart failure. Int J Cardiol.

[108] Schierbeck LL, Jensen TS, Bang U, Jensen G, Kober L, Jensen JE (2011). Parathyroid hormone and vitamin D--markers for cardiovascular and all-cause mortality in heart failure. Eur J Heart Fail.

[109] Pfeffer PE, Chen YH, Woszczek G (2015). Vitamin D enhances production of soluble ST2, inhibiting the action of IL-33. J Allergy Clin Immunol.

[110] Gruson D, Ferracin B, Ahn SA, Rousseau MF (2016). Soluble ST2, the vitamin D/PTH axis and the heart: new interactions in the air?. Int J Cardiol.

[111] Masson S, Barlera S, Colotta F (2016). A low plasma 1,25(OH)(2) vitamin D/PTH (1-84) ratio predicts worsening of renal function in patients with chronic heart failure. Int J Cardiol.

[112] Jiang WL, Gu HB, Zhang YF, Xia QQ, Qi J, Chen JC (2016). Vitamin D supplementation in the treatment of chronic heart failure: a meta-analysis of randomized controlled trials. Clin Cardiol.

[113] Robbins J, Petrone AB, Gaziano JM, Djoussé L (2016). Dietary vitamin D and risk of heart failure in the Physicians' Health Study. Clin Nutr.

[114] Abu El Maaty MA, Hassanein SI, Gad MZ (2016). Genetic variation in vitamin D receptor gene (Fok1:rs2228570) is associated with risk of coronary artery disease. Biomarkers.

[115] Donneyong MM, Hornung CA, Taylor KC (2015). Risk of heart failure among postmenopausal women: a secondary analysis of the randomized trial of vitamin D plus calcium of the women's health initiative. Circ Heart Fail.

[116] Witte KK, Byrom R, Gierula J (2016). Effects of vitamin D on cardiac function in patients with chronic HF: the VINDICATE study. J Am Coll Cardiol.

[117] Schroten NF, Ruifrok WP, Kleijn L (2013). Short-term vitamin D3 supplementation lowers plasma renin activity in patients with stable chronic heart failure: an open-label, blinded end point, randomized prospective trial (VitD-CHF trial). Am Heart J.

[118] Nolte K, Herrmann-Lingen C, Platschek L (2019). Vitamin D deficiency in patients with diastolic dysfunction or heart failure with preserved ejection fraction. ESC Heart Fail.

[119] Ozdemir B (2020). Association between left ventricle ejection fraction and vitamin D levels in congestive heart failure; a cross sectional study. J Pharm Care.

[120] Pandit A, Mookadam F, Boddu S (2014). Vitamin D levels and left ventricular diastolic function. Open Heart.

[121] Liu L, Chen M, Hankins SR (2012). Serum 25-hydroxyvitamin D concentration and mortality from heart failure and cardiovascular disease, and premature mortality from all-cause in United States adults. Am J Cardiol.

[122] Fan Y, Yang Z, Wang L (2023). Traditional Chinese medicine for heart failure with preserved ejection fraction: clinical evidence and potential mechanisms. Front Pharmacol.

[123] Judd SE, Tangpricha V (2009). Vitamin D deficiency and risk for cardiovascular disease. Am J Med Sci.

[124] Boxer RS, Kenny AM, Schmotzer BJ, Vest M, Fiutem JJ, Piña IL (2013). A randomized controlled trial of high dose vitamin D3 in patients with heart failure. JACC Heart Fail.

[125] Dalbeni A, Scaturro G, Degan M, Minuz P, Delva P (2014). Effects of six months of vitamin D supplementation in patients with heart failure: a randomized double-blind controlled trial. Nutr Metab Cardiovasc Dis.

[126] Morillo CA, Banerjee A, Perel P, Wood D, Jouven X (2017). Atrial fibrillation: the current epidemic. J Geriatr Cardiol.

[127] Freedman B, Hindricks G, Banerjee A (2020). World heart federation roadmap on atrial fibrillation—a 2020 update. Glob Heart.

[128] Hu YF, Chen YJ, Lin YJ, Chen SA (2015). Inflammation and the pathogenesis of atrial fibrillation. Nat Rev Cardiol.

[129] Galea R, Cardillo MT, Caroli A, Marini MG, Sonnino C, Narducci ML, Biasucci LM (2014). Inflammation and C-reactive protein in atrial fibrillation: cause or effect?. Tex Heart Inst J.

[130] Patel D, Druck A, Hoppensteadt D, Bansal V, Brailovsky Y, Syed M, Fareed J (2020). Relationship Between 25-Hydroxyvitamin D, Renin, and Collagen Remodeling Biomarkers in Atrial Fibrillation. Clin Appl Thromb Hemost.

[131] Zhang Z, Yang Y, Ng CY, Wang D, Wang J, Li G, Liu T (2016). Meta-analysis of vitamin D deficiency and risk of atrial fibrillation. Clin Cardiol.

[132] Norman PE, Powell JT (2014). Vitamin D and cardiovascular disease. Circ Res.

[133] Demir M, Uyan U, Melek M (2014). The effects of vitamin D deficiency on atrial fibrillation. Clin Appl Thromb Hemost.

[134] Chen WR, Liu ZY, Shi Y, Yin DW, Wang H, Sha Y, Chen YD (2014). Relation of low vitamin D to nonvalvular persistent atrial fibrillation in Chinese patients. Ann Noninvasive Electrocardiol.

[135] Rienstra M, Cheng S, Larson MG (2011). Vitamin D status is not related to development of atrial fibrillation in the community. Am Heart J.

[136] Alonso A, Misialek JR, Michos ED (2016). Serum 25-hydroxyvitamin D and the incidence of atrial fibrillation: the atherosclerosis risk in communities (ARIC) study. Europace.

[137] Özsin KK, Sanrı US, Toktaş F, Kahraman N, Yavuz Ş (2018). Effect of plasma level of vitamin D on postoperative atrial fibrillation in patients undergoing isolated coronary artery bypass grafting. Braz J Cardiovasc Surg.

[138] Gode S, Aksu T, Demirel A, Sunbul M, Gul M, Bakır I, Yeniterzi M (2016). Effect of vitamin D deficiency on the development of postoperative atrial fibrillation in coronary artery bypass patients. J Cardiovasc Thorac Res.

[139] Liu X, Wang W, Tan Z, Zhu X, Liu M, Wan R, Hong K (2019). The relationship between vitamin D and risk of atrial fibrillation: a dose-response analysis of observational studies. Nutr J.

[140] Cerit L, Özcem B, Cerit Z, Duygu H (2018). Preventive effect of preoperative vitamin D supplementation on postoperative atrial fibrillation. Braz J Cardiovasc Surg.

[141] Belen E, Aykan AC, Kalaycioglu E, Sungur MA, Sungur A, Cetin M (2016). Low-level vitamin D is associated with atrial fibrillation in patients with chronic heart failure. Adv Clin Exp Med.

[142] Surdu AM, Pînzariu O, Ciobanu DM (2021). Vitamin D and its role in the lipid metabolism and the development of atherosclerosis. Biomedicines.

[143] Dziedzic EA, Przychodzeń S, Dąbrowski M (2016). The effects of vitamin D on severity of coronary artery atherosclerosis and lipid profile of cardiac patients. Arch Med Sci.

[144] Lupton JR, Faridi KF, Martin SS, Sharma S, Kulkarni K, Jones SR, Michos ED (2016). Deficient serum 25-hydroxyvitamin D is associated with an atherogenic lipid profile: the very large database of lipids (VLDL-3) study. J Clin Lipidol.

[145] Manousopoulou A, Al-Daghri NM, Garbis SD, Chrousos GP (2015). Vitamin D and cardiovascular risk among adults with obesity: a systematic review and meta-analysis. Eur J Clin Invest.

[146] Mirhosseini N, Rainsbury J, Kimball SM (2018). Vitamin D supplementation, serum 25(OH)D concentrations and cardiovascular disease risk factors: a systematic review and meta-analysis. Front Cardiovasc Med.

[147] Asbaghi O, Kashkooli S, Choghakhori R, Hasanvand A, Abbasnezhad A (2019). Effect of calcium and vitamin D co-supplementation on lipid profile of overweight/obese subjects: a systematic review and meta-analysis of the randomized clinical trials. Obes Med.

[148] Sidabutar E, Halim B, Siregar MFG, Lutan D, Adenin I, Kaban Y, idabutar E (2016). Vitamin D levels in women with polycystic ovary syndrome. KnE Med.

[149] Gokosmanoglu F, Onmez A, Ergenç H (2020). The relationship between Vitamin D deficiency and polycystic ovary syndrome. Afr Health Sci.

[150] Miao CY, Fang XJ, Chen Y, Zhang Q (2020). Effect of vitamin D supplementation on polycystic ovary syndrome: a meta-analysis. Exp Ther Med.

[151] Charoenngam N, Holick MF (2020). Immunologic effects of vitamin D on human health and disease. Nutrients.

[152] Manson JE, Cook NR, Lee IM (2019). Vitamin D supplements and prevention of cancer and cardiovascular disease. N Engl J Med.

[153] Hsia J, Heiss G, Ren H (2007). Calcium/vitamin D supplementation and cardiovascular events. Circulation.

[154] Dalan R, Liew H, Assam PN (2016). A randomised controlled trial evaluating the impact of targeted vitamin D supplementation on endothelial function in type 2 diabetes mellitus: the DIMENSION trial. Diab Vasc Dis Res.

[155] Aslanabadi N, Jafaripor I, Sadeghi S (2018). Effect of vitamin D in the prevention of myocardial injury following elective percutaneous coronary intervention: a pilot randomized clinical trial. J Clin Pharmacol.

[156] Hin H, Tomson J, Newman C (2017). Optimum dose of vitamin D for disease prevention in older people: BEST-D trial of vitamin D in primary care. Osteoporos Int.

[157] Neale RE, Armstrong BK, Baxter C (2016). The D-Health trial: a randomized trial of vitamin D for prevention of mortality and cancer. Contemp Clin Trials.

[158] Legarth C, Grimm D, Krüger M, Infanger M, Wehland M (2019). Potential beneficial effects of vitamin D in coronary artery disease. Nutrients.

[159] Seibert E, Lehmann U, Riedel A (2017). Vitamin D(3) supplementation does not modify cardiovascular risk profile of adults with inadequate vitamin D status. Eur J Nutr.

[160] Gholami F, Moradi G, Zareei B, Rasouli MA, Nikkhoo B, Roshani D, Ghaderi E (2019). The association between circulating 25-hydroxyvitamin D and cardiovascular diseases: a meta-analysis of prospective cohort studies. BMC Cardiovasc Disord.

[161] Barbarawi M, Kheiri B, Zayed Y (2019). Vitamin D supplementation and cardiovascular disease risks in more than 83,000 individuals in 21 randomized clinical trials: a meta-analysis. JAMA Cardiol.

[162] McNally JD, Menon K (2013). Vitamin D deficiency in surgical congenital heart disease: prevalence and relevance. Transl Pediatr.

[163] Gallo S, Comeau K, Vanstone C (2013). Effect of different dosages of oral vitamin D supplementation on vitamin D status in healthy, breastfed infants: a randomized trial. JAMA.

[164] Hollis BW, Wagner CL, Drezner MK, Binkley NC (2007). Circulating vitamin D3 and 25-hydroxyvitamin D in humans: an important tool to define adequate nutritional vitamin D status. J Steroid Biochem Mol Biol.

[165] Schlingmann KP, Kaufmann M, Weber S (2011). Mutations in CYP24A1 and idiopathic infantile hypercalcemia. N Engl J Med.

[166] Morris CA, Braddock SR (2020). Health care supervision for children with Williams syndrome. Pediatrics.

[167] Ammenti A, Pelizzoni A, Cecconi M, Molinari PP, Montini G (2009). Nephrocalcinosis in children: a retrospective multi-centre study. Acta Paediatr.

[168] Mantan M, Bagga A, Virdi VS, Menon S, Hari P (2007). Etiology of nephrocalcinosis in northern Indian children. Pediatr Nephrol.

[169] Atabek ME, Pirgon O, Sert A (2006). Oral alendronate therapy for severe vitamin D intoxication of the infant with nephrocalcinosis. J Pediatr Endocrinol Metab.

[170] Chambellan-Tison C, Horen B, Plat-Wilson G (2007). Severe hypercalcemia due to vitamin D intoxication. Arch Pediatr.

[171] Mallet E, Philippe F, Castanet M, Basuyau JP (2010). Administration of a single Winter oral dose of 200,000 IU of vitamin D3 in adolescents in Normandy: evaluation of the safety and vitamin D status obtained (Article in French). Arch Pediatr.

[172] Emel T, Doğan DA, Erdem G, Faruk O (2012). Therapy strategies in vitamin D deficiency with or without rickets: efficiency of low-dose stoss therapy. J Pediatr Endocrinol Metab.

[173] Martineau AR, Jolliffe DA, Hooper RL (2017). Vitamin D supplementation to prevent acute respiratory tract infections: systematic review and meta-analysis of individual participant data. BMJ.

[174] Aggarwal G, Cheruiyot I, Aggarwal S (2020). Association of cardiovascular disease with coronavirus disease 2019 (COVID-19) severity: a meta-analysis. Curr Probl Cardiol.

[175] Zhou F, Yu T, Du R (2020). Clinical course and risk factors for mortality of adult inpatients with COVID-19 in Wuhan, China: a retrospective cohort study. Lancet.

[176] Driggin E, Madhavan MV, Bikdeli B (2020). Cardiovascular considerations for patients, health care workers, and health systems during the COVID-19 pandemic. J Am Coll Cardiol.

[177] Beltrán-García J, Osca-Verdegal R, Pallardó FV (2020). Oxidative stress and inflammation in COVID-19-associated sepsis: the potential role of anti-oxidant therapy in avoiding disease progression. Antioxidants (Basel).

[178] Kox M, Waalders NJ, Kooistra EJ, Gerretsen J, Pickkers P (2020). Cytokine levels in critically ill patients with COVID-19 and other conditions. JAMA.

[179] Aygun H (2020). Vitamin D can prevent COVID-19 infection-induced multiple organ damage. Naunyn Schmiedebergs Arch Pharmacol.

[180] Meltzer DO, Best TJ, Zhang H, Vokes T, Arora V, Solway J (2020). Association of vitamin D status and other clinical characteristics with COVID-19 test results. JAMA Netw Open.

[181] Ebadi M, Montano-Loza AJ (2020). Perspective: improving vitamin D status in the management of COVID-19. Eur J Clin Nutr.

[182] Yancy CW (2020). COVID-19 and African Americans. JAMA.

[183] Mohan M, Cherian JJ, Sharma A (2020). Exploring links between vitamin D deficiency and COVID-19. PLoS Pathog.

[184] Biricik E, Güneş Y (2015). Vitamin D and anaesthesia. Turk J Anaesthesiol Reanim.

[185] Quraishi SA, Bittner EA, Blum L, McCarthy CM, Bhan I, Camargo CA Jr (2014). Prospective study of vitamin D status at initiation of care in critically ill surgical patients and risk of 90-day mortality. Crit Care Med.

[186] Li Y, Ding S (2020). Serum 25-Hydroxyvitamin D and the risk of mortality in adult patients with Sepsis: a meta-analysis. BMC Infect Dis.

[187] Rech MA, Hunsaker T, Rodriguez J (2014). Deficiency in 25-hydroxyvitamin D and 30-day mortality in patients with severe sepsis and septic shock. Am J Crit Care.

[188] Turan A, Hesler BD, You J (2014). The association of serum vitamin D concentration with serious complications after noncardiac surgery. Anesth Analg.

[189] Joshi A, Bhadade R, Varthakavi PK, DeSouza R, Bhagwat NM, Chadha MD (2014). Vitamin D deficiency is associated with increased mortality in critically ill patients especially in those requiring ventilatory support. Indian J Endocrinol Metab.

[190] Aranow C (2011). Vitamin D and the immune system. J Investig Med.

[191] Smith JM, Cancienne JM, Brockmeier SF, Werner BC (2021). Vitamin D deficiency and total shoulder arthroplasty complications. Shoulder Elbow.

[192] Amrein K, Papinutti A, Mathew E, Vila G, Parekh D (2018). Vitamin D and critical illness: what endocrinology can learn from intensive care and vice versa. Endocr Connect.

[193] Haugen M, Brantsaeter AL, Trogstad L, Alexander J, Roth C, Magnus P, Meltzer HM (2009). Vitamin D supplementation and reduced risk of preeclampsia in nulliparous women. Epidemiology.

[194] Nair P, Venkatesh B, Center JR (2018). Vitamin D deficiency and supplementation in critical illness—the known knowns and known unknowns. Crit Care.

[195] Holick MF (2008). The vitamin D deficiency pandemic and consequences for nonskeletal health: mechanisms of action. Mol Aspects Med.

[196] Bolland MJ, Grey A, Avenell A, Gamble GD, Reid IR (2011). Calcium supplements with or without vitamin D and risk of cardiovascular events: reanalysis of the Women's Health Initiative limited access dataset and meta-analysis. BMJ.

[197] Mosekilde L (2008). Vitamin D and the elderly. Clin Endocrinol.

